# Supplementary material for: Poor glycaemic control contributes to a shift towards prothrombotic and antifibrinolytic state in pregnant women with type 1 diabetes mellitus
Source: PLoS One. 2020 Oct 8;15(10):e0237843. doi: 10.1371/journal.pone.0237843 (PMC7544075; doi:10.1371/journal.pone.0237843)
Supplement: Attachment — Submitted filename: rebuttal letter.docx [file pone.0237843.s002.docx]

Dear Reviewer,

We are very grateful for your review. We would like to thank you for a very valuable and matter-of-fact review and for your suggestions of a minor comments. We believe we had addressed very carefully every one of them.

Ad.1 (Please describe the term T80-T10 in the methods)

Response: please find attached file with a revised manuscript track changes. From the revised manuscript: “T80-T10 – time of growth of the thrombus on the AR chip, (time between time of blood clot formation initiation and occlusion time). And more later: “T80-T10 is the interval between T10 and OT or T80 and represents the rate of growth of a thrombus.”

Ad. 2. (Is T1 Is T10 the time it takes to reach 10% of maximum pressure or is it defined different? Please add it to the methods.)

Response: please find attached file with a revised manuscript track changes. From the revised manuscript: “ T10 is defined as the time of the onset of a thrombus formation. It resembles the duration of the flow pressure from baseline values to the 10 kPa pressure values. . It is related with a partial occlusion of microcapillary. ”

Ad. 3. (Figure 4 needs representative traces of the actual T-TAS system to inform the reader of the shape and progression of the curves, particularly because the OT and T80-10 is not significantly different between groups.)

Response:

In our paper we showed that T-TAS® results substantially differed between the group with

sufficient and the group with poor glycemic control. Blood clot formation initiation time was significantly shortened in poorly controlled type 1 diabetic pregnant patients.

There have been numerous scientific data with T-TAS® usage. However in those studies patients were suffering from a heart diseases, were older and had different metabolic diseases. Basically there has been no such research in a pregnant women group at all do date, and moreover no any data in Type 1 Diabetic pregnant patients. We find that notion both innovation and an incentive to a further haemostasis investigation in poor controlled Type 1 Diabetic pregnant women on a larger groups of patients. Moreover we would like to articulate in scientific press a notion of altered whole blood thrombogenicity in poor controlled diabetic patients and thus encourage other researchers to carry out a similar research, not only in a pregnant patients but in the other groups of patients suffering from this disease.

So unfortunately, due to above-mentioned notions, we cannot add any other representative traces of actual TTAS system in pregnancy to fig. No 4 , because our research is the only one on this subject to date; and the only traces that can be compared with a study (diabetes) groups is our healthy pregnant well-matched control group. We hope our research paper will be a stimulus to a further investigations of a whole blood thrombogenicity in a pregnant women. We also highly believe that this phenomenon demands a further research, while pregnancy significantly increases risk of developing an episode of arterial and, especially, venous thrombosis.

Ad. 4. (The manuscript would benefit from an extra round of proof reading since it contains several grammatical errors throughout.)

Response: extra round of proof reading had been carefully performed, and errors had been corrected throughout.

Yours sincerely,

Authors
